# INFORMAL CARE NETWORKS AMONG ADULTS AGING SOLO

**DOI:** 10.1093/geroni/igac059.1414

**Published:** 2022-12-20

**Authors:** Jane Lowers, Duzhi Zhao, Evan Bollens-Lund, Dio Kavalieratos, Katherine Ornstein

**Affiliations:** Emory University, Atlanta, Georgia, United States; Icahn School of Medicine at Mount Sinai, New York, New York, United States; Icahn School of Medicine at Mount Sinai, New York, New York, United States; Emory University, Atlanta, Georgia, United States; Johns Hopkins, University, Baltimore, Maryland, United States

## Abstract

More than 20% of older adults lacks proximal family caregivers. Yet is it unclear who provides support for this growing population. Using 2015 National Health and Aging Trends Study data for 2,998 community-dwelling Medicare beneficiaries who received help with at least one task (e.g., bathing, shopping, insurance help) in the past month, we identified individuals aging solo (no spouse/partner or children residing in the same state) and determined social and caregiving network size and composition. Compared to married peers with nearby children, adults aging solo (7.6% of sample) had social networks of similar size but greater diversity (i.e., more friends, neighbors). Adults aging solo were significantly more likely to rely on paid help and a wider network of informal caregivers, including distal family, and friends. Social networks can anchor interventions to help adults aging solo prepare for future care needs and inform policies to support informal caregiving.

